# Analyse der Versorgungssituation bei Epiphyseolysis capitis femoris (ECF) in Deutschland

**DOI:** 10.1007/s00132-023-04455-6

**Published:** 2023-11-14

**Authors:** Elea Amann, Michael Schwarze, Yvonne Noll, Henning Windhagen, Kerstin Radtke

**Affiliations:** 1 Department Kinder- und Neuroorthopädie, Orthopädische Klinik, Medizinische Hochschule Hannover (MHH) im DIAKOVERE Annastift, Anna-von-Borries-Str. 1–7, 30625 Hannover, Deutschland; 2 Labor für Biomechanik und Biomaterialien, Orthopädische Klinik, Medizinische Hochschule Hannover (MHH) im DIAKOVERE Annastift, Hannover, Deutschland; 3 Klinisches Studienmanagement der Orthopädischen Klinik, Medizinische Hochschule Hannover (MHH) im DIAKOVERE Annastift, Hannover, Deutschland; 4 Orthopädische Klinik, Medizinische Hochschule Hannover (MHH) im DIAKOVERE Annastift, Hannover, Deutschland

**Keywords:** Femoro-acetabuläres Impingement, Kinder, Osteotomie, Krankenhäuser, Fragebogen, Adolescent coxa vara, Children, Hospitals, Osteotomy, Questionnaire

## Abstract

**Einführung:**

Das Behandlungskonzept der Epiphyseolysis capitis femoris wird nach wie vor kontrovers diskutiert. In der Literatur findet sich bislang insgesamt keine einheitliche Empfehlung für ein therapeutisches Vorgehen. Ziel dieser Studie ist daher die Analyse der Versorgungsrealität von Kindern mit ECF in Deutschland.

**Methodik:**

Basierend auf einem Fragebogen zur ECF-Versorgung, der 2021 an ECF-versorgende Ärzte verschickt wurde, erfolgt die Auswertung der Studie. Im Weiteren erfolgt der Abgleich der erhobenen Versorgungsdaten mit Literaturempfehlungen zur ECF.

**Ergebnisse:**

36 von 47 verschickten Bögen wurden eingeschlossen. Dabei konnte insgesamt kein signifikanter Unterschied in der Versorgung der ECF hinsichtlich der jährlichen Fallzahlen oder der Krankenhausgröße nachgewiesen werden.

**Schlussfolgerung:**

Es zeigt sich insgesamt ein inhomogenes Bild bezüglich der ECF-Versorgung. Nach aktueller Literatur gilt das modifizierte Dunn-Verfahren bislang als die beste Therapieoption für schwer abgerutschte Epiphysen und für Patienten mit chronischer ECF. Dieses im Vergleich zu anderen Versorgungsoptionen technisch schwierige und komplikationsreiche Verfahren kann nicht in jedem Krankenhaus angeboten werden. Eine Registererfassung aus versorgenden Kliniken, eine Mindestmengenregelung sowie der Ausbau von Weiterbildungsmaßnahmen können zur Optimierung der Versorgung beitragen.

**Graphic abstract:**

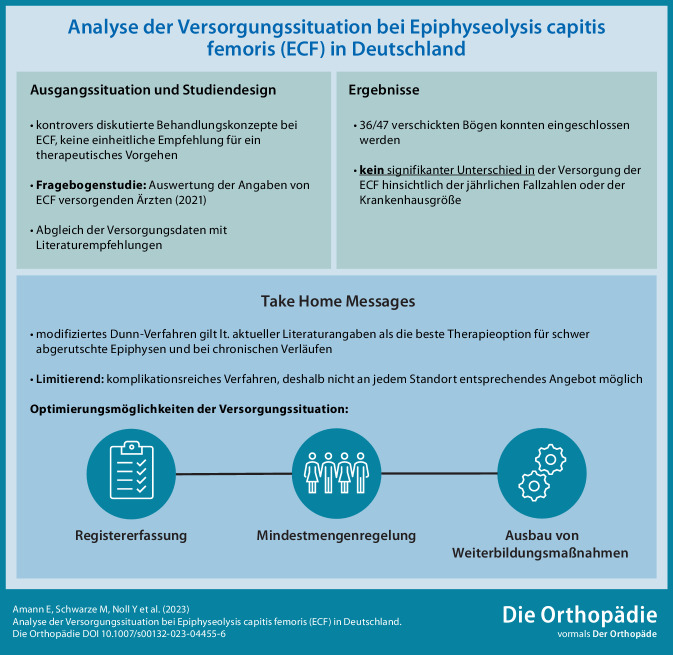

## Abstract (en)

**Introduction:**

The treatment concept for slipped capital femoral epiphysis is still controversial. According to studies, there is currently no recommendation for a universal approach. Therefore, the aim of this study is to analyze the care reality of children with ECF in Germany.

**Methods:**

The evaluation of the study is performed based on a questionnaire sent to physicians tending to ECF in 2021. Data is compared to the literature.

**Results:**

36 of 47 questionnaires sent out were included. Overall, no significant difference in ECF care was proven in terms of annual caseload or the size of hospital.

**Conclusion:**

A high variance in operative SCFE treatment is reported. According to current literature, the modified Dunn procedure is considered the best therapeutic option to date, especially for patients with severe or chronic ECF. However, compared with alternative care options, this is not feasible in every hospital due to its complicating and challenging nature. Central registration, minimum volume regulation, and expansion of continuing education measures can contribute to optimization.

Im Folgenden soll die Versorgungsrealität der ECF genauer dargestellt werden. Auch nötige diagnostische Interventionen, operative Versorgungsmöglichkeiten und Komplikationen sind thematisiert. Abschließend werden Optimierungsstrategien innerhalb eines nationalen Behandlungskonzeptes diskutiert.

## Einführung in das Krankheitsbild der ECF

Bei der ECF kommt es zum Abrutschen der Hüftkopfepiphyse von der Metaphyse nach medial-dorsal-kaudal [20]. Dies geschieht meist ohne vorangehendes Trauma und betrifft vor allem großwüchsige und adipöse Jungen im pubertären Alter [15, 16, 20]. Von der ECF betroffen sind ungefähr 2–10:100.000 Kinder, womit sie die häufigste Hüfterkrankung im Jugendalter darstellt [16].

Während des pubertären Wachstumsschubes kommt es zu physiologischen Veränderungen im Bereich der Hüfte, die die Entstehung einer ECF begünstigen [15, 20]. Die Ätiologie der ECF ist allerdings weiterhin unklar [20].

Die pathologischen Veränderungen differenzieren je nach Symptomdauer der ECF in Abhängigkeit von den Verlaufsformen akut, chronisch oder akut auf chronisch [16, 20]. Die chronische Form tritt dabei mit 75 % am häufigsten auf [20]. Eine Unterteilung erfolgt zusätzlich in Bezug auf den zeitlichen Verlauf des Gleitvorgangs [2]. Tritt die ECF akut auf, erfolgt der Abrutsch plötzlich [7], wohingegen es sich beim langsam fortschreitenden Prozess um die chronische Form handelt [1, 43]. Der Wandel einer chronischen ECF in eine akute Situation erfolgt meist infolge eines inadäquaten Traumas [16]. Neben der Einteilung der ECF nach der Symptomdauer kann die Stabilität und der Schweregrad beurteilt werden [2]. Es wird unterschieden in instabil zu stabil (Stabilität) und in leicht-mäßig-schwer bzw. mild-moderat-schwer (Schweregrad) [2]. Als instabil wird die ECF bezeichnet, sobald dem Patienten eine Belastung, wie die des Auftretens, nicht mehr möglich ist [24]. Die internationale Einteilung nach dem Schweregrad erfolgt anhand des Southwick-Winkels (eingeschlossener Winkel zwischen der Senkrechten zur Epiphysenlinie und der Femurschaftachse) [2, 36]. Alternativ kann auch vom Gesamtepiphysendurchmesser der prozentuale Versatz von Epi- und Metaphyse ermittelt werden [2].

### Klinik der ECF

Anamnestisch können bei bestehender ECF (intermittierende) Schmerzen im Knie‑, Oberschenkel- oder Leistenbereich, neben einer verminderten Belastbarkeit des betroffenen Beines, auffallen [2, 4, 12]. Oft stellt sich das Symptombild der ECF jedoch diffus dar [17]. Typische Merkmale eines ECF-Patienten sind ein eingeschränktes Bewegungsausmaß, besonders in Innenrotation, Flexion und Abduktion, eine grundsätzlich nach außen rotierte und ggf. verkürzte untere Extremität und ein positives Drehmann-Zeichen [4, 12], sodass eine körperliche Untersuchung zur Sicherung der Diagnose unabdingbar ist [2, 20].

### Bildgebende diagnostische Möglichkeiten – Sonographie, Röntgen, MRT und CT

Mittels Sonographie kann ein intraartikulärer Erguss im Hüftgelenk diagnostiziert werden, welcher für die akute und/oder instabile Form des Krankheitsbildes spricht [19].

Diagnostisch ist die Aufnahme von Röntgenbildern in zwei Ebenen grundlegend: eine Beckenübersicht (a. p.) und ein axiales bzw. seitlich axiales Bild beider Hüften entweder nach Lauenstein, als „Cross-table“-Aufnahme oder in „Frog-leg“-Position [2], wobei der axialen Aufnahme mehr Bedeutung zugesprochen wird [15, 47]. Hier ist der Abrutsch nach dorsal, die Schenkelhalsverschiebung nach ventral, sowie die Darstellung der Wachstumsfuge expliziter zu erkennen [2, 15], sodass auch eine Klassifikation anhand der Symptomdauer erfolgen kann [11]. Bei bevorstehendem oder bereits beginnendem Abrutsch kann zur Aufdeckung einer ECF imminens oder incipiens ebenso ein Röntgenbild zur Abklärung hinzugezogen werden [2, 32]. Auffälligkeiten im Bereich der Wachstumsfuge sind hier dann typisch [2, 32]. Die Röntgenaufnahme bildet darüber hinaus zusätzlich die (noch) nicht symptomatische Hüftseite hinsichtlich eines möglichen bilateralen Verlaufes ab [26]. Charakteristisch für eine ECF ist im Röntgenbild unter anderem der Verlust der Schenkelhalstaille und ein Höhenverlust der Epiphyse [11, 15]. Anhand der Röntgenbilder können der Epiphysen-Diaphysen- und Epiphysentorsions-Winkel bestimmt werden, welche zur Stellung der Operationsindikation genutzt werden [11].

Zur weiteren Diagnosesicherung kann zusätzlich eine MRT-Aufnahme hinzugezogen werden [2]. Ein erkennbares Ödem kann hier hinweisend auf einen drohenden Abrutsch sein [2, 21, 41]. Ebenso kann eine beginnende oder chronische Form der ECF eindeutiger detektiert werden [3]. Neben der Möglichkeit der Prüfung der präoperativen Durchblutungssituation im Femurkopf können exaktere Winkelbestimmungen vorgenommen werden [2, 6, 29, 39, 48]. Letzteres ist vorrangig im Rahmen der Indikationsstellung von Repositionen und Korrekturosteotomien wichtig [2]. Postoperativ können Informationen im Hinblick auf mögliche Komplikationen gewonnen werden [2, 27, 38].

Alternativ kann eine Computertomographie zur postoperativen Beurteilung hinsichtlich einer Fehllage eingebrachter Materialien [35] oder innerhalb der Operationsplanung einer Korrekturosteotomie [28] veranlasst werden.

### Therapieoptionen bei ECF

Die Therapie der ECF erfolgt ausschließlich operativ [15, 20, 32]. Zur Auswahl stehen dabei die Fixierung der Wachstumsfuge mit Drähten oder Nägeln, gleitenden Teleskopschrauben und nichtgleitenden, versteifenden Schrauben [22]. Bei Bedarf kann auch zusätzlich reponiert werden [15]. Weiterhin ist eine Umstellung der Hüfte zur Korrektur möglich (z. B. Korrekturosteotomie nach Imhäuser oder modifiziertes Dunn-Verfahren) [8, 15]. So soll ein weiteres Abrutschen gestoppt und die anatomische Rekonstruktion ermöglicht werden [20].

Das Therapieverfahren wird in Abhängigkeit von der Schwere des Abrutsches, des Patientenalters, der Gelenkfunktion, des Nutzen-Risiko-Verhältnisses und der Compliance ausgewählt [22, 32]. Vorrangig orientiert sich die Wahl der Versorgungsoption aber an den ermittelten Abrutschwinkeln in Kombination mit der Akuität der Symptome [15].

Eine prophylaktische Mitversorgung auf der Gegenseite wird in Zentraleuropa mehrheitlich empfohlen, da sich der Gleitvorgang der Epiphysenfuge häufig auf der kontralateralen Seite wiederholt [8, 22, 34].

Die verschiedenen Versorgungsmöglichkeiten bieten unterschiedliche Vorteile. So ist weiteres longitudinales Längenwachstum nach Fixierung mit Kirschner-Drähten (K-Drähten) oder Gleitschraube, im Gegensatz zur Versorgung mit nichtgleitender Schraube weiterhin möglich [22]. Infolge der Epiphyseodese, die sich durch den Einsatz einer nichtgleitenden Schraube ergibt, profitiert der Patient allerdings von der erhöhten Stabilität [22]. Die Versorgung mit Schrauben bietet grundsätzlich den Vorteil der minimal-invasiven Technik im Vergleich zum Einbringen von K‑Drähten [22].

Eine Behandlung der ECF mittels Korrekturosteotomie kommt vor allem für Patienten mit schweren und/oder chronischen Abrutschen infrage oder auch als sekundäre Versorgungsmöglichkeit, nach unzureichendem Einsatz von Schrauben oder Drähten [20, 32]. Der entscheidende und abgrenzende Vorteil der Korrekturosteotomie mittels Dunn-Verfahren liegt in der Wiederherstellung der anatomischen Gegebenheiten, ohne dabei ein gutes Langzeitergebnis zu gefährden [13]. Wesentlicher Bestandteil dieses Verfahrens ist eine chirurgische Hüftgelenksluxation [10, 46], die zusammen mit einem retinakulären Weichteillappen, bestehend aus Retinakulum und Außenrotatoren, die Blutversorgung der Epiphyse sicherstellt [23]. Ohne Spannung auf das Retinakulum auszuüben, kann so der entstandene Kallus abgetragen werden [23]. Durch den luxierten Hüftkopf wird während der anatomischen Reposition sichergestellt, dass das Retinakulum nicht beschädigt wird [23]. Sie birgt allerdings im Vergleich zur Korrekturosteotomie nach Imhäuser ein höheres Komplikationsrisiko und bedarf hinreichender Übung seitens des behandelnden Chirurgen [13, 40].

### Nachsorge

In Abhängigkeit von dem Therapieverfahren und den regelmäßigen Röntgenkontrollen soll postoperativ die Belastung des Beines über ungefähr 6 Wochen eingeschränkt werden [42]. Das prophylaktisch versorgte Bein darf unmittelbar nach erfolgter Fixierung voll belastet werden [42]. Komplikationen treten infolge verspäteter Diagnosestellung [8] oder als Folge therapeutischer Interventionen bei fehlerhaft eingebrachtem Fixiermaterial oder Repositionsmanövern auf [8, 15]. Kurzfristig kann es zur Chondrolyse oder avaskulären Nekrose (AVN) des Hüftkopfes kommen, längerfristig besteht die Gefahr eines femoroazetabulären Impingements (FAI) oder einer sekundären Koxarthrose [22]. Besonders betroffen von (kurzfristigen) Komplikationen sind Patienten, die einen akuten, instabilen oder hochgradigen Abrutsch der Epiphysenfuge erlitten haben [18, 25].

## Zugrundeliegende Methodik der durchgeführten Studie

**Figure 1:**
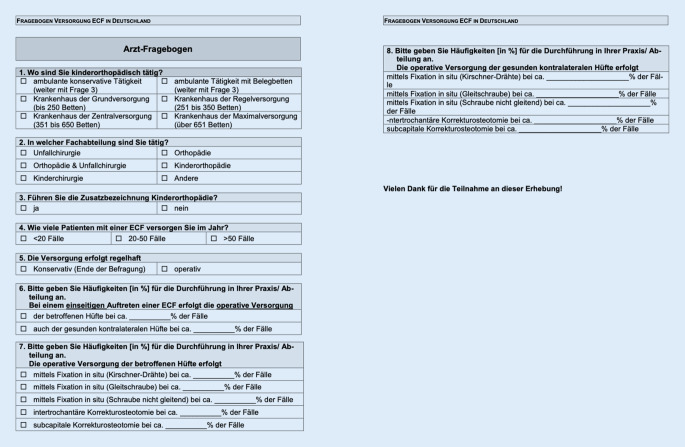

Basierend auf der in der Zeitschrift *Focus Gesundheit *veröffentlichten „Ärzteliste Kinder*“* [9] wurden Fragebögen, die die ECF-Versorgung der behandelten Patienten betreffen, an die aufgeführten Ärzte versendet. Dieser ist in Abb. 1 aufgeführt. Die Liste umfasst 47 in der ECF-Versorgung tätige Kinderorthopäden innerhalb Deutschlands. Die Zusammensetzung der Liste erfolgte dabei auf der Grundlage von Empfehlungen durch ärztliche Kollegen und Patienten. Die kontaktierten Behandler wurden angehalten, Angaben in Bezug auf ihre Versorgungsstufe, jährliche Fallzahlen und die prozentuale Verteilung der operativen Versorgung zu machen (Abb. 1). Es handelt sich bei der Studie demzufolge um eine retrospektive, multizentrische Studie. Die Daten wurden pseudonymisiert erhoben und in einer Excel-Tabelle im klinischen Studienmanagement zusammengetragen und statistisch ausgewertet. Dazu wurden exakte Tests nach Fisher in R mittels der Oberfläche R Studio durchgeführt, das Signifikanzniveau betrug dabei 0,05 [37]. Zur Veranschaulichung der Daten wurden Histogramme mit dem Paket ggplot für R erstellt [44].

### Schwerpunkte des Fragebogens

In acht Frageblöcken wurden die Ärzte zunächst gebeten, Angaben zu den eigenen Gegebenheiten und der Umsetzung der Versorgung zu machen. Berücksichtigt wurden dabei der Bereich der kinderorthopädischen Tätigkeit, die Fachabteilung, in der der interviewte Arzt tätig ist, das Vorliegen der Zusatzbezeichnung Kinderorthopädie, die jährlichen Versorgungsfälle, die standardmäßige Art der Versorgung, die prophylaktische Versorgung bei einseitigem Auftreten, die Häufigkeiten der operativen Versorgungstechnik und auch die Häufigkeiten der operativen Versorgungstechnik zur Vorsorge (Abb. 1).

## Auswertung

Es wurden 36 von 47 versendeten Fragebögen korrekt ausgefüllt und anonym zurückgesendet, sodass sie in die Auswertung eingeschlossen werden konnten. Insgesamt konnte die Studie dabei aber keine signifikanten Unterschiede in der Versorgung der Betroffenen in Abhängigkeit von den jährlichen Fallzahlen (oder der Versorgungsstufe) des therapierenden Klinikums nachweisen.

## Ergebnisse

**Figure 2:**
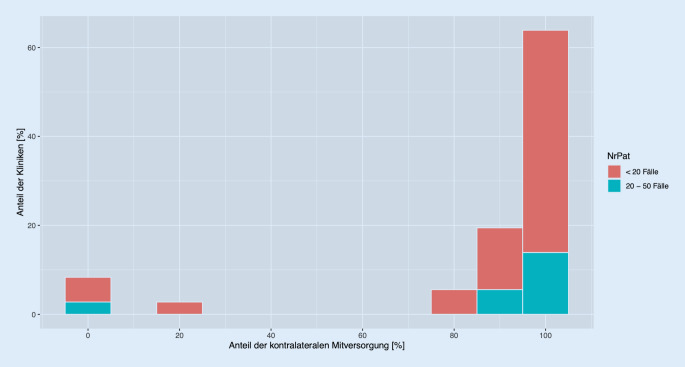

Jeweils knapp ein Drittel der Befragten (27,8 % bzw. 10/36) ist den Angaben zufolge innerhalb der Grund- oder Maximalversorgung tätig (Tab. 1). Mit ca. 5–15 % sind die übrigen Teilnehmenden ambulant oder einem Krankenhaus der Regel- oder Zentralversorgung tätig. Dabei tragen alle Befragten die Zusatzbezeichnung Kinderorthopäde. Insgesamt nahmen weniger Kollegen an der Studie teil, die in einem Krankenhaus mit höherer Bettenzahl (Zentral- und Maximalversorger: ab 351 Betten) arbeiten. Die Mehrheit der Befragten behandelt eine geringere Menge an Patienten mit ECF. Dabei geben 77,8 % eine Versorgung von weniger als 20 Patienten im Jahr an. Die restlichen 22,2 % notieren die Versorgung von 20–50 Fällen jährlich. Grundsätzlich therapieren alle Befragten eine ECF operativ. Die prophylaktische Versorgung erfolgt bei dem größten Anteil der Befragten: rund 85 % geben an, in 80–100 % der Fälle die gesunde Seite zu fixieren (Abb. 2).

**Table 1:** 

Ort der Tätigkeit	Relativer (in %) und absoluter Anteil
Ambulante konservative Tätigkeit	5,6 (2)
Ambulante Tätigkeit mit Belegbetten	13,9 (5)
Krankenhaus der Grundversorgung (< 250 Betten)	27,8 (10)
Krankenhaus der Regelversorgung (251–350 Betten)	11,1 (4)
Krankenhaus der Zentralversorgung (351–650 Betten)	13,9 (5)
Maximalversorgung (ab 651 Betten)	27,8 (10)

**Figure 3:**
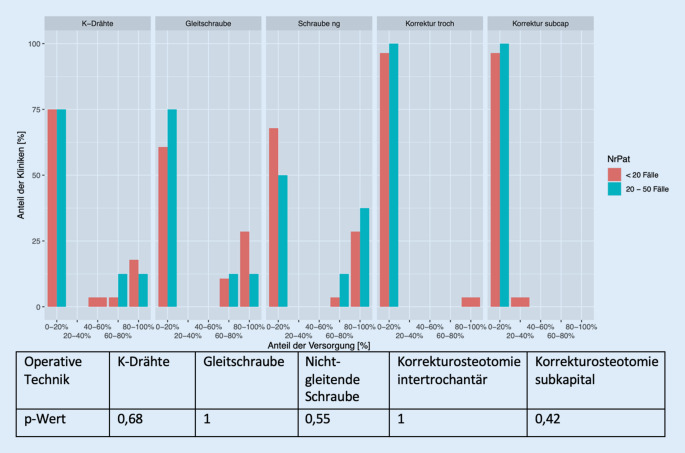

In Abhängigkeit von den Fallzahlen kann Folgendes festgehalten werden: Die Kliniken, die angeben weniger als 20 Fälle jährlich zu versorgen haben, bevorzugen die Behandlung mit Schrauben (gleitende/nichtgleitende). Kliniken mit mehr als 20 Fällen verwenden eher nichtgleitende Schrauben. Korrekturosteotomien werden sehr zurückhaltend mit gar nicht bis wenig (0–20 %) angegeben. Der Vergleich zwischen Kliniken mit mehr und weniger als 20 Fällen hinsichtlich der Verwendung der gängigen Versorgungsverfahren bei betroffener Hüfte zeigte keine Unterschiede (K-Drähte [*p* = 0,68], Gleitschraube [*p* = 1], nichtgleitende Schraube [*p* = 0,55], Korrekturosteotomie intertrochantär [*p* = 1], Korrekturosteotomie subkapital [*p* = 0,42]) (Abb. 3).

**Figure 4:**
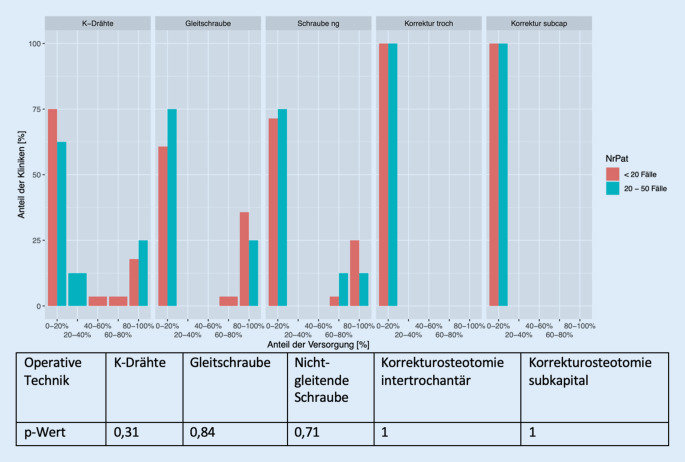

Im Hinblick auf die prophylaktische Versorgung zeigt sich bezogen auf die Fallzahlen, dass Kliniken mit weniger als 20 zu versorgenden Fällen zum Einsatz der Gleitschraube tendieren, Kliniken mit 20–50 Fällen pro Jahr geben zu ähnlichen Anteilen die Gleitschraube und K‑Drähte an. Wie auch hinsichtlich der Versorgung der betroffenen Seite zu beobachten ist, wird ebenso innerhalb der Behandlung der kontralateralen Hüftseite kein signifikanter Unterschied nachgewiesen (K-Drähte [*p* = 0,31], Gleitschraube [*p* = 0,84], nichtgleitende Schraube [*p* = 0,71], Korrekturosteotomie intertrochantär [*p* = 1], Korrekturosteotomie subkapital [*p* = 1]) (Abb. 4).

**Figure 5:**
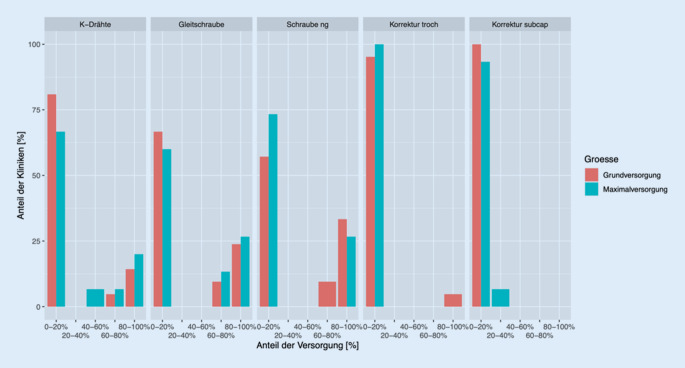

**Figure 6:**
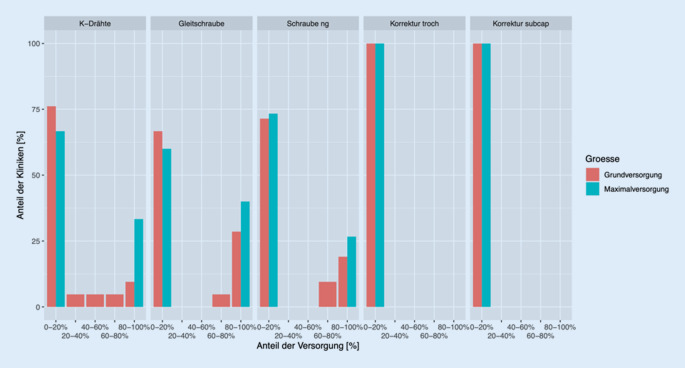

Unter Berücksichtigung der Klinikgröße ist zu konstatieren, dass Kollegen, die innerhalb der Zentral- und Maximalversorgung tätig sind, vorrangig Schrauben (gleitend/nichtgleitend) bei der Versorgung der betroffenen Seite verwenden. Ärzte, die in kleineren Kliniken (unter 351 Betten) behandeln, bevorzugen den Einsatz von nichtgleitenden Schrauben (Abb. 5). Die kontralaterale Seite wird unabhängig von der Klinikgröße, vorrangig mit einer Gleitschraube versorgt. Korrekturosteotomien werden laut der Teilnehmenden zur prophylaktischen Therapie nicht angewendet (Abb. 6).

## Einordnung der Ergebnisse

Auch wenn diese Arbeit keine signifikanten Ergebnisse zeigen konnte, fallen dennoch Tendenzen und Aspekte innerhalb der ausgewerteten Befragung auf. Da hier fallzahlenbedingte Effekte nicht auszuschließen sind, wäre die Untersuchung eines größeren Studienkollektivs zukünftig wünschenswert. Es ergibt sich ein inhomogenes Bild für die Anwendung der unterschiedlichen Versorgungsmethoden (Abb. 3 und 5). Damit deckt sich auch die aktuelle Literatur, in der es heißt, dass es bisher im Hinblick auf die Überlegenheit eines der Fixationsimplantate noch keine Evidenz gebe [22]. Weiterhin werde die Wahl des verwendeten Materials unter Berücksichtigung der Erfahrung und des Kenntnisstandes des Operateurs getroffen [22]. Lederer et al. [22] empfehlen außerdem nichtgleitende Schrauben nur eingeschränkt zur grundsätzlichen Versorgung der ECF [22]. Vorrangig sollte sie bei älteren Kindern eingebracht werden, da diese sich voraussichtlich nicht mehr lange in der Wachstumsphase befinden [22]. Im Fragebogen ist das Alter der behandelten Patienten nicht erfragt worden, sodass hinsichtlich dieses Aspektes keine Einordung erfolgen kann.

Einseitig von ECF betroffene Patienten sind einem erhöhten Risiko ausgesetzt ebenfalls einen Abrutsch auf der kontralateralen Hüftseite zu erfahren. Deshalb wird in Zentraleuropa mehrheitlich die prophylaktische Versorgung empfohlen. Dies kann so auch in dieser Fragebogenstudie beobachtet werden. Es geben rund 85 % der Teilnehmenden an, bei 80–100 % der vorstellig werdenden Jugendlichen eine vorsorgliche Fixierung der Gegenseite durchzuführen.

Zur prophylaktischen Behandlung der gesunden Hüfte wird unter anderem die Verwendung einer Gleitschraube oder die Verwendung von K‑Drähten empfohlen, denn diese können das longitudinale Wachstum weiterhin gewährleisten [14, 22]. Die Befragten geben zur prophylaktischen Behandlung vorrangig die Gleitschraube an: Versorger mit einer Bettenanzahl unter 351 notieren, anteilig über 25 % ihrer Patienten in 80–100 % der Fälle mit einer Gleitschraube zu versorgen. Zentral- und Maximalversorger tendieren mit über 37,5 % anteilig in 80–100 % der Fälle zur Gleitschraube. Kliniken mit Fallzahlen zwischen 20 und 50 geben zu gleichen Anteilen von 25 % an, K‑Drähte oder eine Gleitschraube zu verwenden. Auch Krankenhäuser mit Fallzahlen bis 20 nannten die Gleitschraube als häufigste angewandte Versorgungsmethode zur Fixierung der kontralateralen Hüfte mit einem Anteil von mehr als 30 % in 80–100 % der Fälle. Die in der Literatur ausgesprochene Empfehlung stimmt hier mit dem am häufigsten angegebenen Anteil der ausgewerteten Ergebnisse überein.

Im Hinblick auf die Versorgung der betroffenen Seite fiel eine Bevorzugung der Behandlung durch eine Fixation grundsätzlich im Vergleich zu Korrekturosteotomien auf. Dass Korrekturosteotomien laut der Abfrage anteilig insgesamt wenig durchgeführt werden, lässt sich auf verschiedene Gründe zurückführen. Zum einen sind Korrekturosteotomien grundsätzlich eher zur Versorgung schwerer chronischer Abrutsche ab einem Abrutschwinkel von 60 ° (teilweise auch schon ab 30 °) vorgesehen, zum anderen gilt eine Korrekturosteotomie, besonders die nach dem Dunn-Verfahren, als technisch deutlich komplexer und komplikationsreicher im Vergleich zu anderen Versorgungsoptionen [20, 32, 40, 46]. Eine intertrochantäre Korrekturosteotomie wird häufig erst als Reaktion auf eine eingetretene Folgekomplikation der ECF, einer AVN, durchgeführt [8]. Dadurch kann die Belastung auf die entstandenen Nekroseareale verringert werden [8].

**Figure 7:**
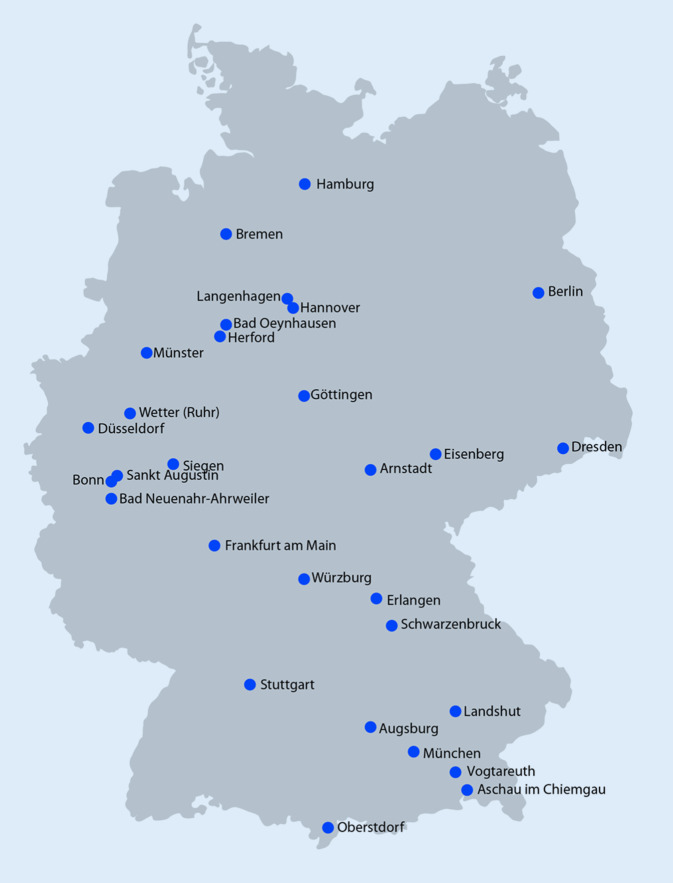

In der aktuellen Literatur wird vor allem die Dunn-Operation, eine subkapitale Korrekturosteotomie, als die bestmögliche Therapieoption bei chronischen Verläufen beschrieben [42]. Angaben aus der Literatur zufolge, sind durchschnittlich 75 % der ECF-Erkrankten von einem chronischen Verlauf betroffen [20]. Ist die Stichprobe an Patienten, die durch die befragten Kollegen versorgt wurden, repräsentativ für das gesamte Patientenkollektiv innerhalb von Deutschland, stellt sich die Frage, ob diese Patienten womöglich von der komplexeren Dunn-Korrekturosteotomie profitiert hätten. Auch wurde die Versorgung durch Korrekturosteotomien nach Imhäuser nur in sehr geringem Maße angegeben. In der Literatur wird diese als komplikationsärmer und von der Durchführung als einfacher beurteilt als die Dunn-Osteotomie [40]. Da die teilnehmenden Kollegen ihre Standorte innerhalb von ganz Deutschland haben (Abb. 7), und diese Kollegen ebenso auf der Grundlage von Empfehlungen befragt wurden, ist von einer repräsentativen Patientengruppe auszugehen. Im Zuge des Fragebogens wurde jedoch nicht ermittelt, wie die Patienten innerhalb ihrer Erkrankung klassifiziert wurden (z. B. akut/chronisch, stabil/instabil). Diesbezüglich lässt sich deshalb keine genaue Aussage treffen und sollte in einer weiterführenden Untersuchung betrachtet werden.

Innerhalb der Angaben über die Durchführung einer Korrekturosteotomie konnte jedoch beobachtet werden, dass eine subkapitale Dunn-Osteotomie generell eher von größeren Kliniken, wie Zentral- und Maximalversorgern, durchgeführt wird. Ca. 5 % geben an, anteilig rund 30–40 % der Fälle mit dieser Operationsmethode zu therapieren. Kleinere Kliniken geben eine Rate von 0–20 % an. Jedoch notieren diese wiederum, im Gegensatz zu den größeren Kliniken, in ca. 5 % der Fälle zu 80–100 % ihre Patienten mit einer Korrekturosteotomie nach Imhäuser zu behandeln. Unter Berücksichtigung der Fallzahlen von weniger als 20 im Jahr bzw. 20–50 jährlichen Fällen, konnte diese Beobachtung jedoch nicht bestätigt werden. Hier gaben lediglich die Befragten mit geringerer jährlicher Fallzahl die Durchführung von Korrekturosteotomien nach Imhäuser an, nicht aber die Kliniken mit höheren Fallzahlen die Versorgung mittels Dunn-Verfahren. So stimmt das ermittelte Ergebnis nur bedingt mit der aktuellen Literatur überein. In dieser wird vor allem den Kliniken mit hohen Fallzahlen die Durchführung des Dunn-Verfahrens empfohlen, da diese ein gutes Training des Chirurgen voraussetzt [13]. Die Größe der Klinik kann, muss aber nicht unbedingt mit den jährlichen Fallzahlen korrelieren. Aufgrund der Notwendigkeit einer entsprechenden Übung mit dem Dunn-Verfahren gibt es so bisher keine standardisierte Empfehlung für die Durchführung [13]. Dies spiegeln auch die ermittelten Ergebnisse des Fragebogens durch die sehr geringen anteiligen Angaben für dieses Operationsverfahren wider.

Nach der aktuellen Befragung werden Korrekturosteotomien nur selten durchgeführt. Auf Basis der aktuellen Erkenntnisse wird für Patienten mit schweren und/oder chronischen Abrutschen jedoch eine subkapitale Korrekturosteotomie empfohlen. Insbesondere von geübten Chirurgen sollte dies demzufolge vermehrt durchgeführt werden, um dem Patienten ein bestmögliches Outcome zu ermöglichen.

Die Epiphyseolysis capitis femoris ist mit einer Inzidenz von 2–10:100.000 die häufigste Hüftpathologie im Kindes- und Jugendalter. Hinzu kommt, dass seit 1980 eine steigende Tendenz der Erkrankungsfälle zu beobachten ist [30]. Auch die Corona-Pandemie wird voraussichtlich ihren Teil dazu beitragen: Die Deutsche Gesellschaft für Kinder- und Jugendmedizin (DGKJ) berichtete zuletzt von einer Steigerung der Adipositasrate bei Kindern und Jugendlichen [33]. Neben einer verminderten femoralen Antetorsion gilt die Adipositas als wichtigster Risikofaktor für die Entwicklung einer ECF [31], sodass auch eine weitere Steigerung an ECF-Fällen zu erwarten sein könnte.

Darüber hinaus veröffentlichten Wirth et al. kürzlich eine Arbeit, die zeigt, dass das Fachgebiet der Kinderorthopädie vergleichsweise unterrepräsentiert ist [45]. Hier heißt es, „es ist davon auszugehen, dass in etwa 30 % der deutschen Universitätskliniken kein kinder- und jugendorthopädisches Angebot vorgehalten wird“ [45]. Zudem gebe es Defizite in Lehre und Forschung [45]. Diese Erkenntnis, zusammen mit dem Fakt, dass besonders die chronische Form der ECF, aufgrund seines meist diffusen Symptombildes, immer noch gehäuft verspätet diagnostiziert wird, spricht für den dringenden Ausbau von Weiterbildungsmaßnahmen im kinderorthopädischen Fachgebiet.

Sinnhaft könnte zusätzlich eine Mindestmengenregelung sein, wie sie auch schon innerhalb der Endoprothetik angewandt wird [5]. Besonders die Möglichkeit der Versorgung der ECF mittels Dunn-Verfahren spricht aufgrund seines komplexen und komplikationsreichen Charakters ebenso dafür.

Betrachtet man im Hinblick darauf noch einmal die Verteilung der befragten Kollegen innerhalb von Deutschland, ist grundsätzlich auch eine Bündelung der Kliniken im Westen und Südosten Deutschlands erkennbar (Abb. 7). Besonders in unterrepräsentierten Gebieten wäre eine Zentrumsbildung denkbar, um eine bessere Versorgungsqualität gewährleisten zu können.

## Fazit für die Praxis

- Es besteht die Notwendigkeit des Ausbaus von Weiterbildungsmaßnahmen im Fachgebiet Kinderorthopädie.
- Die zentrale Registererfassung der Versorgungsrealität für einzelne Krankheitsbilder wie die Epiphyseolysis capitis femoris (ECF) führt zur besseren Beurteilung der nationalen Versorgungslage.
- Eine Zentralisierung von kinderorthopädischen Versorgungsstandorten könnte sinnvoll sein, um zukünftig die Qualität der Behandlung von Kindern mit ECF zu steigern.
- Die Einführung einer Mindestmengenregelung für komplexere Eingriffe wie das Dunn-Verfahren sollte diskutiert werden.

## Abkürzungsverzeichnis

a.p.: Aterio-posteriorer Strahlengang
CT: Computertomographie
ECF: Epiphyseolysis capitis femoris
FAI: Femoro-acetabuläres Impingement
MRT: Magnet-Resonanz-Tomographie
SCFE: Slipped capital femoral epiphysis
